# Hippocampal and Amygdala Gray Matter Loss in Elderly Controls with Subtle Cognitive Decline

**DOI:** 10.3389/fnagi.2017.00050

**Published:** 2017-03-07

**Authors:** Davide Zanchi, Panteleimon Giannakopoulos, Stefan Borgwardt, Cristelle Rodriguez, Sven Haller

**Affiliations:** ^1^Department of Psychiatry, University of BaselBasel, Switzerland; ^2^Department of Neuropsychiatry, University Psychiatry ClinicBasel, Switzerland; ^3^Department of Psychiatry, Faculty of Medicine, University of GenevaGeneva, Switzerland; ^4^Affidea Carouge Radiologic Diagnostic Center, GenevaSwitzerland; ^5^Department of Surgical Sciences, Radiology, Uppsala University, UppsalaSweden; ^6^Department of Neuroradiology, University Hospital FreiburgFreiburg, Germany; ^7^Department of Neuroradiology, Faculty of Medicine of the University of GenevaGeneva, Switzerland

**Keywords:** hippocampus, amygdala, Alzheimer, FSL FIRST, MCI, cognitive impairment, deteriorating controls

## Abstract

In contrast to the idea that hippocampal and amygdala volume loss occur in late phases of neurodegeneration, recent contributions point to the relevance of preexisting structural deficits that are associated with aging and are independent of amyloid deposition in preclinical Alzheimer disease cases. The present work explores GM hippocampal and amygdala volumes in elderly controls displaying the first signs of cognitive decline. 455 subjects (263 females), including 374 controls (228 females) and 81 middle cognitive impairment subjects (35 females), underwent two neuropsychological evaluations (baseline and 18 months follow-up) and a MRI-T1 examination (only baseline). Clinical assessment included Mini-Mental State Examination (MMSE), Clinical Dementia Rating scale, Hospitalized Anxiety and Depression scale, the Consortium to Establish a Registry for Alzheimer’s Disease neuropsychological battery and RI-48 Cued Recall Test (RI-48) for episodic memory. Based on their cognitive performance, we defined the controls as stable controls (sCON) and deteriorating controls (dCONs). Analyses included volumetric assessment, shape analyses and linear regressions between GM volume loss and differences in clinical scores between baseline and follow-up. Significant GM volume decrease in hippocampus bilaterally and right amygdala was found in dCON compared to sCON (*p* < 0.05). Lower right amygdala volumes were measured in mild cognitive impairment (MCI) compared to sCON (*p* < 0.05). Shape analyses revealed that atrophy was more pronounced at the superior- posterior lateral side of the hippocampus and amygdala. Significant correlations were found between GM volume of left hippocampus and the delta of MMSE and RI-48 scores in dCON and MCI groups separately. Decreased hippocampal and right amygdala volumes precede the first signs of cognitive decline in healthy elderly controls at the pre-MCI state. Left hippocampus volume may also predict short-term changes of overall cognition in these vulnerable cases.

## Introduction

The first neuropathological hallmarks described by Alois Alzheimer more than a century ago, amyloid deposits and neurofibrillary tangles (NFTs), are present in hippocampal subdivisions and restricted neocortical areas of cognitively normal people and their densities increase with age (Giannakopoulos et al., 2007). Progressively, AD pathogenesis has been perceived as a long lasting morbid process, with an extensive prodromal phase. Subsequent advent of modern non-invasive diagnostic techniques enabled early detection of diverse AD-related pathological events *in vivo*, and prepared the ground for the notion of preclinical AD (Lazarczyk et al., 2012). This concept refers to asymptomatic stages of AD that are characterized by the progressive development of functional and subsequently structural changes in cortical areas in the absence of clinically overt signs of the disease. Early appearance of various biological changes suggestive of AD pathology in controls has been extensively documented. They may precede clinically overt dementia by many years, or even decades (Reiman et al., 2004; Shaw et al., 2007; Lazarczyk et al., 2012), thus defining a temporally wide preclinical phase of the disease (Lazarczyk et al., 2012).

The appearance of these preclinical markers has been described as a highly ordered process summarized by Jack et al. (2010) in their sequential model of the evolution of AD. According to this model, the earliest AD markers are those related to amyloid deposits in Positron emission tomography (PET) with Pittsburg compound B (Fagan et al., 2006; Forsberg et al., 2010), followed by increased cerebrospinal fluid (CSF) tau and phospho-tau levels (Fagan et al., 2009), brain hypometabolism in FDG- PET (18-Fluorodeoxy-glucose PET), and abnormal activation pattern in functional magnetic resonance imaging (fMRI) upon cognitive task solving (Liang et al., 2011; Miao et al., 2011). Initially, very subtle morphological brain alterations were thought to become detectable in structural magnetic resonance imaging (MRI) only in later phases of neurodegeneration in preclinical AD, notably, a decrease in entorhinal cortex, hippocampal and amygdala volume (Miller et al., 2013; Younes et al., 2014).

A recently published work on 1209 cognitively intact individuals (Jack et al., 2015) showed that hippocampal volume loss may occur before abnormal amyloid PET occurrence pointing to the relevance of preexisting structural deficits that are associated with aging and are independent on the current amyloid pathology-determined definition of preclinical AD. Similar observations were reported by Edmonds indicating that neurodegeneration alone was 2.5 times more common than amyloidosis alone among healthy individuals who subsequently progress to mild cognitive impairment (MCI) (Edmonds et al., 2015).

The current investigation aims to extend the established findings of GM loss in the earliest stage of cognitive decline before MCI or even AD. We assessed of 455 elderly individuals (mean age, 73.5 ± 4.2 years, 263 females) including notably 374 healthy controls with intact cognitive status at inclusion. Based on neurocognitive follow-up at 18 months about half of controls remained cognitively stable (sCON), while the other half had subtle cognitive deteriorating control (dCON). It is important to emphasize, that even the dCON participants at follow-up were still in the range of normal cognitive performance, yet with respect to the baseline assessment, these participants expressed first subtle cognitive deficits, clearly less pronounced to qualify for an MCI state. We explored the patterns of GM loss in hippocampus and amygdala in these elderly controls displaying the first signs of cognitive decline as compared to stable controls as well as MCI cases as a second group of comparison.

## Materials and Methods

### Participants

The protocol was approved by the ethics committee of the University Hospitals of Geneva. All experimental procedures were carried out in accordance with the approved guidelines and with the principles of the Declaration of Helsinki. All participants were given written informed consent prior to inclusion. Participants were contacted via advertisements in local media to guarantee a community-based sample. An experienced neuropsychologist (C.R.) clinically assessed all individuals independently. Those who met dementia DSM-IV diagnostic criteria on the basis of the neuropsychological and clinical assessments were excluded. Moreover, exclusion criteria included psychiatric or neurologic disorders, sustained head injury, history of major medical disorders (neoplasm or cardiac illness). Moreover, subjects were included if they had no alcohol or drug abuse, no regular use of neuroleptics, antidepressants or psychostimulants and no contraindications to MR imaging. To control for the confounding effect of cardiovascular diseases, individuals with subtle cardiovascular symptoms and a history of stroke and transient ischemic episodes were also excluded from the present study. The inclusion period for control subjects and patients with MCI was from October 2010 to March 2016.

The final sample included 455 elderly individuals (mean age, 73.5 ± 4.2 years, 263 females). Among them, 182 were subsequently classified in the sCON group (mean age, 73.1 ± 3 years, 112 females), 192 in the dCON group (74.2 ± 4.1 years, 116 females), and 81 in the MCI group (73.9 ± 5 years, 35 females).

### Neuropsychological Assessment

At baseline (T1), all individuals underwent neuropsychological assessment. The control and MCI participants were evaluated with an extensive neuropsychological battery, including:

(a) Mini-Mental State Examination (MMSE) (Folstein et al., 1975): the most often used short screening tool to provide an overall measure of the cognitive impairment of the subjects (Arevalo-Rodriguez et al., 2015);
(b) Hospitalized Anxiety and Depression scale (HAD) (Zigmond and Snaith, 1983): self-assessment scale has been developed and found to be a reliable instrument for detecting states of depression and anxiety in the setting of an hospital medical outpatient clinic;
(c) Consortium to Establish a Registry for Alzheimer’s Disease (CERAD) battery (Welsh et al., 1994): standardized battery to assess various manifestations of Alzheimer’s disease.
(d) Clinical Dementia Rating scale (CDR) (Hughes et al., 1982): clinical scale to investigate the staging of dementia.
(e) RI-48 Cued Recall Test (RI-48) (Buschke et al., 1997): clinical test to investigate memory impairment.

Education level was defined according to the Swiss scholar system: level 1, less than 9 years (primary school); level 2, between 9 and 12 years (high school); and level 3, more than 12 years (university). Moreover, only cases with a CDR score of 0 and scores within 1.5 standard deviations of the age-appropriate mean in all other tests were included in the control group.

Participants with a CDR score of 0.5 but no dementia and a score more than 1.5 standard deviations below the age-appropriate mean in any of the previously mentioned tests were confirmed to have MCI, in agreement with the criteria of Petersen et al. (2001).

Eighteen months (±2 weeks) after the baseline evaluation (T2), control subjects underwent cognitive reassessment with the same neuropsychological battery. Participants were placed in the dCON group at follow-up if they had a performance 0.5 standard deviation lower than that at inclusion for two or more neuropsychological tests. Additionally, all individuals were clinically assessed independently by two board certified neuropsychologists (S.T., E.T.; 4 and 2 years of experience, respectively). The final classification of dCON was made blindly by a trained neuropsychologist (C.R., 10 years of experience) using both the neuropsychological scores and clinical assessment. For the extensive neuropsychological evaluation and clinical tests scores at T1 see Supplementary Material and previously published works (Xekardaki et al., 2015).

### APOE Assessment

Whole blood samples were collected at baseline for all subjects for APOE genotyping. Standard DNA extraction was performed using either 9 ml EDTA tubes (Sarstedt, Germany) or Oragene Saliva DNA Kit (DNA Genotek, Inc., Ottawa, ON, Canada) which were stored at -20°C. APOE genotyping was done on the LightCycler (Roche Diagnostics, Basel, Switzerland) as described previously (Nauck et al., 2000). Subjects were divided based on if they were a carrier of the APOEε4 allele (ε4/ε3, ε3/ε3, ε3/ε2 carrier).

### MRI Imaging

All participants underwent MRI examination including two structural (T1 and T2) sequences.

Images were obtained using a 3T scanner (Trio; Siemens, Erlangen, Germany) with a standard 32-channel head-coil. A magnetization-prepared rapid gradient-echo three-dimensional T1-weighted sequence was performed for spatial normalization and gray matter segmentation with the following fundamental parameters: 2300/2.3, 256 × 256 matrix, 176 sections, and 1 mm × 1 mm × 1 mm.

Additional sequences included axial fast spin-echo T2-weighted imaging (4000/105, 30 sections, 4-mm section thickness), susceptibility weighted imaging (28/20, 208 × 256 × 128 matrix, 1 mm × 1 mm × 1 mm voxel size) were performed to exclude brain disease, such as ischemic stroke, subdural hematomas, or space-occupying lesions.

### Assessment of Gray Matter Volumes

FMRIB’s Integrated Registration and Segmentation Tool (FSL FIRST^1^) was applied according to the standard procedure (de Jong et al., 2008; Patenaude et al., 2011) to estimate left and right gray matter volumes of the hippocampus and amygdala. FIRST is part of FMRIB’s Software Library (FSL) and performs registration and segmentation of these regions (Patenaude et al., 2011). The 3D T1 images are registered into MNI (Montreal Neurological Institute) standard space, by means of affine transformations based on 12 degrees of freedom. After registration, FSL-FIRST applies *ad hoc* masks to locate the structures by segmentation based on shape models and voxel intensities. ROIs were defined using the Harvard-Oxford Subcortical Structural Atlas implemented in FSL. The absolute volumes were calculated, taking into account the transformations made in the first stage. Subsequently, a boundary correction was used to determine which boundary voxels belong to the structure or not. In this study a Z-value of 3 was used, corresponding to a 99.998% certainty that the voxel belonged to the subcortical structure. All segmented regions were visually checked for errors in registration and segmentation.

Brain tissue volume was estimated with SIENAX^2^ (Smith et al., 2002), part of FSL (Smith et al., 2004). SIENAX extracts brain and skull images from the single raw input data (Smith et al., 2002; Woolrich et al., 2009). Tissue-type segmentation with partial volume estimation was carried out to calculate total volume of brain tissue (including volumes of gray matter, white matter). For this study we used the absolute volumes generated by the algorithm. Intracranial volume was calculated by adding the volumes of cerebral spinal fluid, total gray matter and total white matter.

After extracting the volumes of the identified areas (FIRST and SIENAX-output) a one-way ANOVA group comparison was performed including GM volumes as dependent variable, and age, gender, educational level, APOE genotype and intracranial volume as non-explanatory covariates. Tukey corrections for multiple comparison was applied with a *p* < 0.05 threshold.

Moreover, after FIRST, vertex analysis was performed using the standard procedure (Patenaude et al., 2011) and shape changes were assessed on a per vertex basis. GM atrophy in the vertices across all subjects was assessed using a general linear model (GLM) with age as covariate using permutation-based non-parametric testing (Randomize, part of FSL). Threshold-free cluster enhancement (TFCE) multiple comparison correction was applied, considering corrected *p*-values < 0.05 as significant.

### Statistical Analysis

Group differences in age, gender, end education as well as changes in neuropsychological scores between T1 and T2 were assessed using a one-way independent ANOVA with Tukey correction for *post hoc* pair-wise comparisons. Correlations between neuropsychological scores changes (delta T1–T2) and GM volumes at baseline were assessed using Spearman rank correlation coefficients. In addition, linear regression models were built with the delta of cognitive scores as dependent variable and MRI parameters at baseline, age, years of education, and APOE genotyping as independent variables. Of note, the variable gender was not considered in this linear regression since a separate general regression analysis showed no differences in cognitive performance between males and females within each diagnostic group. Statistical analyses were performed using GraphPad Prism (Version 6, GraphPad Software, San Diego, CA, USA), R (Version 0.99.896, The R Project for Statistical Computing) FSL (Version 5.0.6, FMRIB, Oxford, UK).

## Results

### Demographic, Genetic, and Neuropsychological Testing at T2

The dCON cases were slightly older than MCI cases (*p* < 0.05) with no age differences between sCON and the other two diagnostic groups [*F*(2,452) = 4.34, η^2^ = 0.2, *p* < 0.05]. A lower proportion of women was found in MCI group (43%) compared to sCON (61%) (*p* < 0.01) and dCON (60%) [*F*(2,452) = 4.34, η^2^ = 0.02, *p* < 0.05]. Education level [*F*(2,452) = 2.42, η^2^ = 0.01, *p* = 0.32] and APOE allele distribution did not differ between the three groups [*F*(2,452) = 4.34, η^2^ = 0.02, *p* = 0.25].

Upon follow-up, higher MMSE scores were found in sCON compared to dCON (*p* < 0.001) and MCI (*p* < 0.001) and in dCON group compared to MCI cases (*p* < 0.01) [*F*(2,452) = 26.54, η^2^ = 0.11, *p* < 0.001]. Moreover, significantly lower HAD scores [*F*(2,452) = 5.16, η^2^ = 0.02, *p* < 0.01] were found in sCON compared to MCI cases (*p* < 0.01). As expected, dCON cases showed lower CDR scores compared to MCI cases (*p* < 0.001) [*F*(2,452) = 1.6, η^2^ = 0.02, *p* < 0.001]. RI-48 performance was lower in MCI compared to dCON (*p* < 0.001) and sCON (*p* < 0.001) and in dCON compared to MCI (*p* < 0.001) [*F*(2,452) = 65, η^2^ = 0.3, *p* < 0.001]. Finally, significantly higher CERAD scores were found in sCON compared to MCI (*p* < 0.05) [*F*(2,452) = 3.1, η^2^ = 0.05, *p* < 0.05] (**Table 1**). For the results of the neuropsychological assessment at T1, see Supplementary Material and Supplementary Table A.

**Table 1:** Demographic, genetic, and neuropsychological results at follow-up.

	sCON	dCON	MCI			*p*-value	
	(182)	(192)	(81)	Group	sCON vs. dCON	sCON vs. MCI	dCON vs. MCI
Age	73.1 ± 3.8	74.2 ± 4.1	73.1 ± 3	*p* < 0.05 η^2^ = 0.2	/	/	*p* < 0.05
Sex	61% f	60% f	43% f	*p* < 0.01 η^2^ = 0.02	/	*p* < 0.01	*p* < 0.01
Education	2.2 ± 0.7	2.1 ± 0.7	2.2 ± 0.6	/ η^2^ = 0.01	/	/	/
APOE	17.5% ε4	20.3%% ε4	16% ε4	/ η^2^ = 0.02	/	/	/
MMSE	28.6 ± 1.1	27.9 ± 1.4	27.1 ± 2.2	*p* < 0.001 η^2^ = 0.11	*p* < 0.001	*p* < 0.001	*p* < 0.01
HAD	5.5 ± 3.4	6.1 ± 3.8	7.3 ± 4.3	*p* < 0.01 η^2^ = 0.02	/	*p* < 0.01	/
CDR	/	0.1 ± 0.1	0.4 ± 0.2	*p* < 0.001 η^2^ = 0.02	/	/	*p* < 0.001
RI-48	29.7 ± 5.4	26.3 ± 6.1	18.3 ± 6.9	*p* < 0.001 η^2^ = 0.3	*p* < 0.001	*p* < 0.001	*p* < 0.001
CERAD	10.8 ± 0.8	10.6 ± 0.9	10.4 ± 1.7	*p* < 0.05 η^2^ = 0.05	/	*p* < 0.05	/


*Mean and standard deviations are shown. η^*2*^ refers to eta-squared effect size of the ANOVA. sCON, stable controls; dCONs, deteriorating controls; MCI, mild cognitive impairment; APOE, apolipoprotein E; MMSE, Mini-Mental State Examination; HAD, Hospitalized Anxiety and Depression scale; CDR, Clinical Dementia Rating scale; RI-48, RI-48 Cued Recall Test; CERAD, Consortium to Establish a Registry for Alzheimer’s Disease.*

**FIGURE 1:**
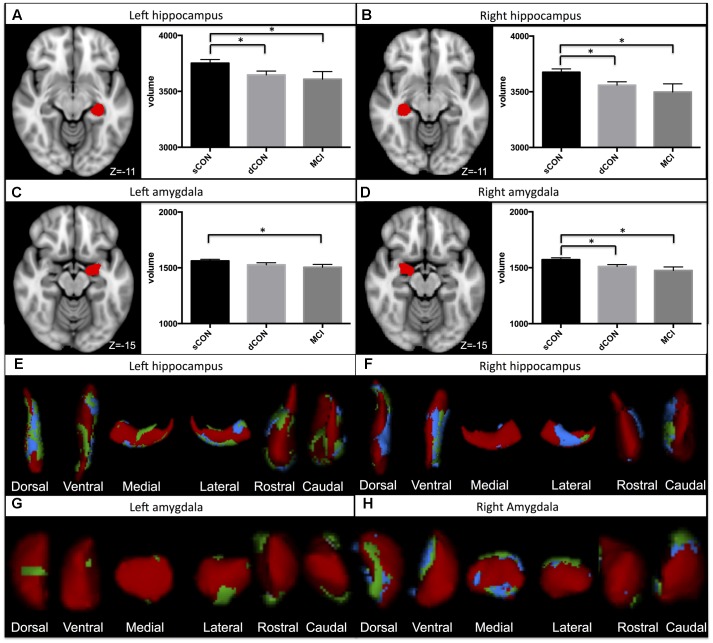
**Significant atrophy was found in the volumes of the left hippocampus, right hippocampus, and right amygdala in stable controls (sCON) compared to deteriorating controls (dCONs) (*p* < 0.05) and mild cognitive impairment (MCI) (*p* < 0.05) **(A,B,D)**.** Significant GM atrophy was found in the volumes of the left amygdala between sCON and MCI (*p* < 0.05) **(C)**. GM loss was more pronounced at the superior- posterior lateral side of both structures. Significant atrophy for dCON compared to sCON was shown in blue, while atrophy for the MCI group compared to sCON was shown in green **(E–H)**. No significant differences were found between dCON and MCI in the considered subcortical GM volumes.

Significant shape differences were found using vertex analyses in the bilateral hippocampus and amygdala. In particular, GM atrophy was more pronounced at the superior- posterior lateral side of the structures both for the dCON compared to sCON and for MCI compared to sCON (**Figures 1E–H**). No significant contribution of age was observed.

### Gray Matter Volumes

Significant differences were found in the volumes of the left hippocampus [*F*(2,452) = 4.51, η^2^ = 0.015, *p* < 0.05], right hippocampus [*F*(2,452) = 4.23, η^2^ = 0.014, *p* < 0.05] and right amygdala [*F*(2,452) = 5.3, η^2^ = 0.02, *p* < 0.01] between sCON and dCON (*p* < 0.05) as well as between sCON and MCI cases (*p* < 0.05) (**Figures 1A,B,D**). Significant differences were found in the volumes of the left amygdala between sCON and MCI cases (*p* < 0.05) [*F*(2,452) = 3.54, η^2^ = 0.01, *p* < 0.05] (**Figure 1C**). No significant differences were found between dCON and MCI in the considered subcortical GM volumes.

In order to address the possible confounding effect of APOE genotype and gender on the observed differences, we performed a one-way ANOVA including GM volumes as dependent variable, and age, gender, educational level, APOE genotype and intracranial volume as non-explanatory covariates. Moreover, we also repeated the analysis inserting in the model the interaction APOExgender as non-explanatory variable. Our data persisted after controlling for APOE and gender. In fact both variables were not associated with GM volumes in the present sample.

### Linear Regressions MRI Results – Clinical Scores

**FIGURE 2:**
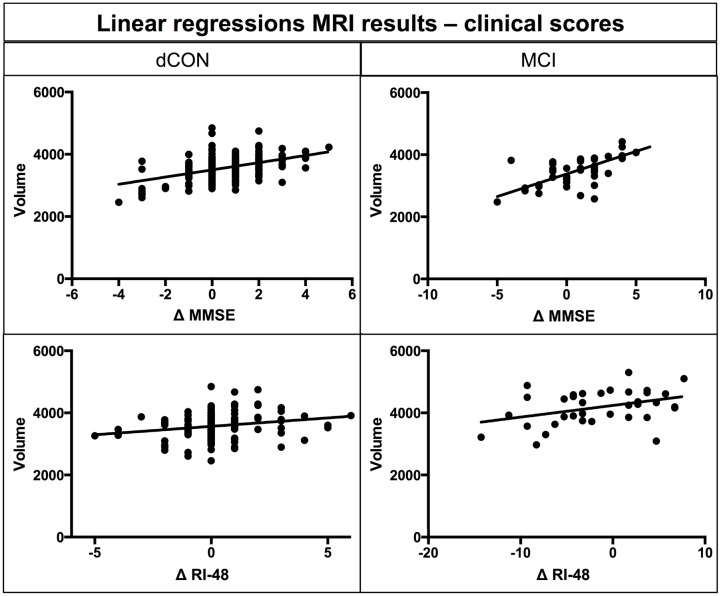
**Linear regression between atrophy in hippocampal volumes at T1 and differences in MMSE and RI-48 performance**.

Significant associations were found between gray matter volume of left hippocampus and delta of MMSE and RI-48 scores in dCON (MSSE: β-coefficient = 0.47, *p* < 0.001; RI-48: β-coefficient = 0.51, *p* < 0.001) and MCI (MSSE: β-coefficient = 0.65, *p* < 0.001; RI-48: β-coefficient = 0.18, *p* < 0.01) groups (**Figure 2**).

## Dicussion

Our results show early atrophy of the bilateral hippocampus and right amygdala that exist already in dCON, a group of elderly controls displaying subtle cognitive deficits at 18 months follow-up compatible with a pre-MCI stage (Jack et al., 2010). A gradient effect is also present with MCI having the highest GM volume loss in these limbic areas and dCON occupying an intermediate position. Our results show that this population is clinically and biologically distinguishable from healthy controls (sCON), confirming previous studies suggesting that GM atrophy in limbic areas predicts subsequent cognitive decline already in an asymptomatic state preceding MCI (Jack et al., 2010; Edmonds et al., 2015).

In mild AD, atrophy has a predilection for brain areas invaded from NFTs. In agreement with this idea, early studies showed that the volume of the medial temporal lobe discriminates controls from both clinically overt AD and MCI cases. This MRI parameter is also associated with time to progress from MCI to AD (Atiya et al., 2003; Kantarci and Jack, 2003; Younes et al., 2014). Previous investigations also indicated that baseline measures of the hippocampus and amygdala in preclinical AD patients predict subsequent development of MCI (Grundman et al., 2002; den Heijer et al., 2006; Griffith et al., 2013; Guderian et al., 2015). Our findings go beyond these data in that they demonstrate that GM volume decrease is present bilaterally in the hippocampus and in the right amygdala. This decrease is more pronounced in the lateral side of the structures in elderly controls that rapidly develop the first signs of cognitive decline prior to MCI. In the same line, Perrotin et al. (2015) and Delli Pizzi et al. (2016) reported that elderly individuals with subjective cognitive decline as well as AD cases showed a major atrophy of the lateral part of the CA1 field of the hippocampus and subiculum. Younes also reported that hippocampal and amygdala GM atrophy occurs 2–4 years prior to the first signs of AD (Younes et al., 2014). However, the GM loss in these areas varies substantially in preclinical AD cases reflecting the heterogeneity of the structural damage at this stage of the degenerative process (Lauriola et al., 2016; Perrotin et al., 2016).

The association between this early structural damage and amyloid pathology is still disputed. In its recent PET/MRI study, Wang et al. (2015) suggested that amyloid pathology associated with preclinical AD mostly affects hippocampal volume. In contrast, Insel et al. (2015) showed that hippocampal atrophy in preclinical AD cases may take place before Aβ positivity. Interestingly, our observations also documented an association between the extent of GM loss in left hippocampus and MMSE and RI-48 scores decrement in dCON and MCI cases suggesting that this MRI parameter may also serve as a marker of cognitive changes in vulnerable cases at risk for AD (Apostolova et al., 2006; Dinomais et al., 2016).

In our multivariate models, the association between lower GM volume values in hippocampus and right amygdala at baseline and dCON status persisted after correcting for possible confounders and in particular APOE4 allele. Previous studies indicated that, among cognitively normal individuals, APOE can lead to hypo-metabolism in posterior cingulate cortex (Reiman et al., 2004), worse cognitive performance (Caselli et al., 2009), and smaller regional brain volumes (Shaw et al., 2007; Alexander et al., 2012). Our results are consistent with the recent work by Jack et al. (2015) showing no effect of APOE gene alleles on memory and cognitive decline and with previous studies showing no association between APOE ε4 and hippocampal volume in cognitively normal individuals (Jack et al., 1998; Protas et al., 2013; Lupton et al., 2016).

### Strengths and Limitations

Strengths of the present study includes its longitudinal design, large numbers of community-dwelling cases, as well as detailed neuropsychological testing at inclusion and follow-up. However, some limitations should also be considered. Consistent with recent core clinical criteria for MCI (Albert et al., 2011), the identification of dCONs was based on the objective decline in cognitive functions measured using serial, comprehensive neuropsychological assessments. However, in the absence of longer follow-up the cognitive fate of these cases remains uncertain so that they cannot be *a priori* considered as incipient AD cases. No CSF measures of tau and Aβ protein were available in this work so that the real extent of AD pathology remains unknown. Future studies exploring the tau and amyloid status based on PET in our community-based cohort are warranted to examine the relationship between AD molecular events and MRI atrophy in limbic areas in dCON.

## Conclusion

The present study focuses on detection of early cognitive deterioration, showing that atrophy of the limbic system (hippocampal and amygdala GM volume loss) is present and predicts decline in cognitive performance in elderly healthy individuals already before MCI.

## Author Contributions

Conceived the study: DZ, SH, PG, SB; recruited: CR, DZ, SH; performed the analyses: DZ, SH, PG, SB, CR; manuscript writing: DZ, SH, PG, SB.

## Conflict of Interest Statement

The authors declare that the research was conducted in the absence of any commercial or financial relationships that could be construed as a potential conflict of interest.

The reviewer ML and handling Editor declared their shared affiliation, and the handling Editor states that the process nevertheless met the standards of a fair and objective review.

## Footnotes

1 http://fsl.fmrib.ox.ac.uk/fsl/fslwiki/FIRST

2 http://fsl.fmrib.ox.ac.uk/fsl/fslwiki/SIENA
