# Supplementary material for: Hippocampal and Amygdala Gray Matter Loss in Elderly Controls with Subtle Cognitive Decline
Source: Front Aging Neurosci. 2017 Mar 7;9:50. doi: 10.3389/fnagi.2017.00050 (PMC5340094; doi:10.3389/fnagi.2017.00050)
Supplement: Supplementary file 1 [file Table_1.DOCX]

**SUPPLEMENTARY MATERIAL**

*Neuropsychological assessment*

At baseline, all individuals were evaluated with an extensive neuropsychological battery, including the Mini-Mental State Examination (MMSE) [1], the Hospital Anxiety and Depression Scale (HAD)[2], and the Lawton Instrumental Activities of Daily Living (IADL)[3]. Cognitive assessment included (a) attention (Digit-Symbol-Coding[4], Trail Making Test A[5], (b) working memory (verbal: Digit Span Forward[4]), visuo-spatial: Visual Memory Span (Corsi)[6], (c) episodic memory (verbal: RI-48 Cued Recall Test) [7] or RL/RI-16 Free and Cued Recall Test, visual: Shapes Test[8], (d) executive functions (Trail Making Test B[5], Wisconsin Card Sorting Test and Phonemic Verbal Fluency Test), (e) language (Boston Naming) [9], (f) visual gnosis (Ghent Overlapping Figures), (g) praxis: ideomotor [10], reflexive [11], and constructional (Consortium to Establish a Registry for Alzheimer’s Disease (CERAD), Figures copy) [12]. All individuals were also evaluated with the Clinical Dementia Rating scale (CDR)[13].

*Results of Neuropsychological assessment at T1*

At T1, higher MMSE scores were found in sCON compared to MCI (p<.05) and in dCON group compared to MCI cases (p<.05) [F(2, 452) = 3.6, η2=.05 , p<.05]. No significant differences were found between the three groups for the HAD test [F(2, 452) = 0.9, η2=.0 , p=0.4], while as expected, dCON cases showed lower CDR scores compared to MCI (p<.001) cases and sCON (p<.001) [F(2, 452) = 1033, η2=.14 p<.001].

No significant differences were found between the three groups for the RI-48 test [F(2, 452) = 1.7, η2=.02 , p=0.2] and finally no significant differences were found between the three groups for the CERAD test [F(2, 452) = 1.1, η2=.02 , p=0.3].

**TABLE A**

|  | sCON | dCON | MCI |  |  | P-value |  |
| --- | --- | --- | --- | --- | --- | --- | --- |
|  | (182) | (192) | (81) | Group | sCONvsdCON | sCONvsMCI | dCONvsMCI |
| MMSE | 28.4 ± 2.3 | 28.3 ± 2.4 | 27.3 ± 3.9 | p<.05 | / | p<.05 | p<.05 |
| HAD | 5.8 ± 3.4 | 6.1 ± 3.8 | 5.7 ± 3.3 | / | / | / | / |
| CDR | / | / | 0.5 ± 0.1 | p<.001 | / | p<.001 | p<.001 |
| RI-48 | 27.5 ± 5.2 | 27.3 ± 5.4 | 15.8± 5.8 | / | / | / | / |
| CERAD | 10.8 ± 0.9 | 10.6 ± 1 | 10.6 ± 1.4 | / | / | / | / |

Table A. Demographic, genetic and neuropsychological results at T1. List of abbreviations: Stable Controls (sCON), Deteriorated Controls (dCON), Mild Cognitive Impairment (MCI), Apolipoprotein E (APOE), Mini-Mental State Examination (MMSE), Hanxiety and Depression Scale (HAD), Clinical Dementia Rating (CDR), RI-48 Cued Recall Test (RI-48), Consortium to Establish a Registry for Alzheimer’s Disease (CERAD).

**REFERENCES**

[1] Arevalo-Rodriguez I, Smailagic N, Roqué I Figuls M, Ciapponi A, Sanchez-Perez E, Giannakou A, Pedraza OL, Bonfill Cosp X, Cullum S (2015) Mini-Mental State Examination (MMSE) for the detection of Alzheimer’s disease and other dementias in people with mild cognitive impairment (MCI). *Cochrane Database Syst. Rev.* CD010783.

[2] Snaith RP (2003) The Hospital Anxiety And Depression Scale. *Health Qual. Life Outcomes* **1**, 29.

[3] Barberger-Gateau P, Commenges D, Gagnon M, Letenneur L, Sauvel C, Dartigues JF (1992) Instrumental activities of daily living as a screening tool for cognitive impairment and dementia in elderly community dwellers. *J. Am. Geriatr. Soc.* **40**, 1129–1134.

[4] Wechsler Adult Intelligence Scale® - Third Edition.

[5] Reitan RM (1958) VALIDITY OF THE TRAIL MAKING TEST AS AN INDICATOR OF ORGANIC BRAIN DAMAGE. *Percept. Mot. Skills* **8**, 271.

[6] Milner B (1971) Interhemispheric differences in the localization of psychological processes in man. *Br. Med. Bull.* **27**, 272–277.

[7] Buschke H, Sliwinski MJ, Kuslansky G, Lipton RB (1997) Diagnosis of early dementia by the Double Memory Test: encoding specificity improves diagnostic sensitivity and specificity. *Neurology* **48**, 989–997.

[8] Doors and People | Pearson Assessment.

[9] Kaplan E, Goodglass H, Weintraub S, Goodglass H (1983) *Boston naming test*, Lea & Febiger, Philadelphia.

[10] Schnider A, Hanlon RE, Alexander DN, Benson DF (1997) Ideomotor apraxia: behavioral dimensions and neuroanatomical basis. *Brain Lang.* **58**, 125–136.

[11] Neuropsychological Studies of Apraxia and Related Disorders, Volume 23 - 1st Edition ISBN: 9780444876690 - 9780080866772.

[12] Welsh KA, Butters N, Mohs RC, Beekly D, Edland S, Fillenbaum G, Heyman A (1994) The Consortium to Establish a Registry for Alzheimer’s Disease (CERAD). Part V. A normative study of the neuropsychological battery. *Neurology* **44**, 609–614.

[13] Hughes CP, Berg L, Danziger WL, Coben LA, Martin RL (1982) A new clinical scale for the staging of dementia. *Br. J. Psychiatry J. Ment. Sci.* **140**, 566–572.
